# Screening Antifungal and Antioxidant Activity of Macroalgae from SE Spain Highlights the Invader *Rugulopteryx okamurae*

**DOI:** 10.3390/plants15101485

**Published:** 2026-05-13

**Authors:** Miguel Valverde-Urrea, Carolina Kaylly Otero, Marc Terradas-Fernández, Federico Lopez-Moya

**Affiliations:** Plant Pathology Laboratory, Department of Marine Sciences and Applied Biology, Universidad of Alicante, Aptdo. Correos 99, 03080 Alicante, Spain; carolinakaylly@gmail.com (C.K.O.); marc.terradas@ua.es (M.T.-F.)

**Keywords:** algae, antioxidants, antifungals, *Rugulopteryx okamurae*, *Batophora* sp., *Fusarium oxysporum*

## Abstract

The Mediterranean Sea harbors a rich diversity of macroalgae with pharmacological potential. In this study, antioxidant and antifungal activities of methanol and ethyl acetate extracts from *Rugulopteryx okamurae*, *Dictyota fasciola*, *Batophora* sp., *Codium fragile*, and *Palisada tenerrima* from the southeastern coast of Spain were evaluated. *R. okamurae*, *Batophora* sp. and *C. fragile* are non-native. All extracts exhibited antioxidant activity, particularly those obtained with methanol. *R. okamurae* and *Batophora* sp. showed the highest activity, inhibiting the DPPH· radical by more than 40% at 1 mg/mL. Methanolic extracts of *R. okamurae* and *P. tenerrima* showed in vitro fungistatic activity against the banana plant pathogenic fungus *Fusarium oxysporum* f. sp. cubense tropical race 4. *R. okamurae* exhibited the highest antifungal activity, with inhibition values of 23.3% and 30.5% at 10 and 20 mg/well, respectively, while *P. tenerrima* showed moderate activity, and the remaining extracts had lower effects. LC–MS/MS analysis of *R. okamurae* revealed a diverse metabolite profile, including oxylipin-type metabolites and terpenoid-related compounds. These results indicate that macroalgae from the southeastern Spanish coast may represent a source of antioxidant and antifungal compounds and support the valorization of invasive biomass.

## 1. Introduction

Marine macroalgae are a rich source of bioactive compounds with promising pharmacological potential. In recent years, growing interest has emerged in exploring algae as natural sources of antioxidants and antifungals, due to their unique secondary metabolite profiles and sustainable availability [1,2]. Among these, species inhabiting the southeastern coast of Spain—including both native and invasive taxa—have drawn attention for their abundant biomass and potential for biotechnological applications.

Invasive macroalgae such as *Rugulopteryx okamurae*, *Codium fragile* or *Batophora* sp. have proliferated along the Mediterranean coast, disrupting ecosystems and local biodiversity [3,4,5]. Beyond their ecological impacts, these alien species represent an untapped biomass resource with potential value for bioactive compound extraction. Using this biomass to obtain antioxidant and antifungal extracts could not only generate value-added products but also contribute to mitigating their environmental impact [6,7]. This ecological pressure highlights the need for valorization strategies, where the biochemical potential of these species can be explored as a source of bioactive compounds.

Several studies have highlighted the antioxidant capacity of marine algal extracts, attributed mainly to their content of phenolic compounds, flavonoids, terpenoids, carotenoids, and other metabolites [8,9]. These include compounds such as fucoxanthin, phlorotannins, and various terpenoid derivatives, which have been widely associated with antioxidant and antimicrobial activities. Antioxidants from marine algae may play an important role in neutralizing reactive oxygen species (ROS), thereby preventing oxidative stress-induced cellular damage—a process implicated in the development of various chronic diseases, including cancer and neurodegenerative disorders [10,11].

In addition to their antioxidant potential, algal extracts have demonstrated significant antifungal activity against phytopathogens such as *Fusarium oxysporum*, including the highly aggressive f.sp. cubense tropical race 4 (TR4), which is responsible for Fusarium wilt in banana crops [12]. Current control measures are limited, and no effective treatment exists for this pathogen, highlighting the need for alternative, sustainable antifungal agents [13,14]. Algal secondary metabolites may offer promising antifungal activities, as demonstrated in previous in vitro studies [15,16].

Extraction methods and solvent polarity are known to influence the recovery of bioactive compounds from algae, with polar solvents such as methanol showing greater efficacy in extracting phenolic and flavonoid compounds, thereby enhancing antioxidant and antifungal activities [17,18].

Given the increasing demand for natural antioxidants and antifungals, coupled with the environmental need to manage invasive algal species, the valorization of marine macroalgae from the southeastern coast of Spain represents a viable strategy for sustainable biotechnological development. *Fusarium oxysporum* f. sp. *cubense* TR4 is a major phytopathogen for which effective control strategies remain limited, highlighting the need for alternative antifungal approaches. In this context, the evaluation of invasive species such as *Rugulopteryx okamurae* against this pathogen remains largely unexplored.

The selected species include both invasive and native macroalgae with ecological relevance and different taxonomic groups. Invasive species such as *R. okamurae*, *C. fragile* and *Batophora* sp. were chosen due to their high biomass availability and environmental impact, while *Dictyota fasciola* was included as a representative native brown alga. In addition, *Palisada tenerrima* was selected as a less-studied red alga to explore its potential as a source of bioactive compounds. We hypothesize that methanolic extracts will exhibit higher antioxidant and antifungal activity than ethyl acetate extracts due to their greater efficiency in extracting polar bioactive compounds, and that brown algae will show higher bioactivity compared to green and red algae due to their distinct metabolite composition.

Therefore, the objective of this study is to evaluate the antioxidant and antifungal activities of methanol and ethyl acetate extracts from selected macroalgae collected from accessible coastal areas of southeastern Spain. We also aim to assess the potential of invasive species biomass for biotechnological applications, thereby contributing to both developing new resources to manage Fusarium wilt in banana and developing sustainable environmental management of algal biomass for practical uses.

## 2. Materials and Methods

### 2.1. Collection and Identification of Algal Species

Algal samplings were carried out in the upper littoral zone (≈0.25 m depth). *R. okamurae* and *C. fragile* were collected from Agua Amarga Beach (SE Spain, 38.299019, −0.519183), *D. fasciola* and *P. tenerrima* from Cabo de las Huertas (SE Spain, 38.353344, −0.414069), and *Batophora* sp. from Punta del Galán, Mar Menor (SE Spain, 37.69006, −0.74234). Collections were carried out in November 2023, except for *Batophora* sp. (March 2023). Samples were kept on ice during transport to prevent degradation.

Algal samples were cleaned of sediments and epiphytes and identified morphologically using specialized keys [5,19,20,21,22,23]. Molecular identification was performed following a CTAB-based DNA extraction protocol [24] with minor modifications. Briefly, freeze-ground algal tissue using liquid nitrogen was incubated in CTAB buffer (0.1 M Tris–HCl, 0.05 M EDTA, 1.5 M NaCl, 0.05 DTT, 2% PVP, 3% CTAB) at 60 °C, followed by chloroform:isoamyl alcohol (24:1, *v*/*v*) extraction and ethanol precipitation. DNA pellets were washed with 70% ethanol, air-dried, and resuspended in nuclease-free water. DNA quality and concentration were assessed using a NanoDrop One C spectrophotometer (Thermo Fisher Scientific, Waltham, MA, USA).

PCR amplifications were performed using VWR Red Taq DNA polymerase Master Mix in a LifeExpress thermal cycler (BIOER, Hangzhou, China). For brown algae, the psbA gene was amplified using primers psbAF1 (5′-ATGACTGCTACTTTAGAAAGAC-3′) and psbAR2 (5′-TCATGCATWACTTCCATACCTA-3′) [25] under a two-step cycling protocol: an initial denaturation (94 °C, 2 min), followed by 5 cycles (94 °C for 30 s, 45 °C for 30 s, and 72 °C for 1 min), and 35 cycles (94 °C for 39 s, 46.2 °C for 39 s, and 72 °C for 1 min), with a final extension at 72 °C for 7 min. Green algae were identified by amplification of the rbcL gene using primers rbcL B (5′-ATGTCACCACAAACAGAAACTAAAGCA-3′) and rbcL Q (5′-GATCTCCTTCCATACTTCACAAGC-3′) [26], with cycling conditions of 94 °C for 3 min; 30 cycles of 94 °C for 1 min, 50 °C for 30 s, and 72 °C for 1.5 min; and a final extension at 72 °C for 7 min. For red and brown algae, the COI gene was amplified using primers GazF2 (5′-CCAACCAYAAAGATATWGGTAC-3′) and GazR2 (5′-GGATGACCAAARAACCAAAA-3′) [27], with an initial denaturation at 94 °C for 4 min, followed by 40 cycles (94 °C for 1 min, 50 °C for 30 s, and 72 °C for 1 min) and a final extension at 72 °C for 7 min.

PCR products were verified on 1% agarose gels, purified using the GeneJET Gel Extraction Kit (Thermo Scientific), and sequenced by Sanger sequencing (STAB Vida, Caparica, Portugal). Resulting sequences were compared against the National Center for Biotechnology Information database using BLASTn.

### 2.2. Algae Extract Preparation

Fresh algal samples were freeze-dried for 48 h in a CHRIST Alpha 1–2 LDplus lyophilizer (Martin Christ Gefriertrocknungsanlagen GmbH, Osterode am Harz, Germany). Moisture content was calculated by weight difference. Dried samples were ground in liquid nitrogen.

Extracts were made by mixing 20 g of powdered algae with methanol or ethyl acetate HPLC grade at a 1:10 (*w*/*v*) ratio [28]. Methanol and ethyl acetate were selected due to their different polarity, allowing for the extraction of a broad range of bioactive compounds. Extraction involved heating at 40 °C under stirring for 2 h, followed by stirring for 48 h at 4 °C. The maceration method was selected as a simple and reproducible approach for comparative screening, and because it was performed at low temperature, it helped preserve thermolabile compounds.

Extracts were filtered through Whatman No.1 filter paper and 0.22 µm syringe filters (Merck KGaA, Darmstadt, Germany), evaporated under reduced pressure at 45 °C using a Büchi R-100 rotary evaporator (Büchi Labortechnik AG, Flawil, Switzerland), and stored at −20 °C. Dried extracts were dissolved in dimethyl sulfoxide (DMSO) to a final stock concentration of 100 mg/mL. Extraction yield was calculated as:

$$Yield\left(\%\right)=\frac{dry\;extract\;weight}{dry\;seaweed\;weight}\times 100$$

Each extraction was performed in technical triplicate and pooled to reduce variability.

### 2.3. Algae Extract Characterization

#### 2.3.1. Total Phenolic Content

Phenolic content was quantified using the Folin–Ciocalteu method [29]. Absorbance was measured at 760 nm, expressed as µg gallic acid equivalents per mg dry extract.

#### 2.3.2. Total Flavonoid Content

Flavonoid content was measured by mixing 0.5 mL extract with 1 mL 10% AlCl_3_ and 0.5 mL 120 mM potassium acetate, incubating for 30 min at 30 °C, and reading absorbance at 415 nm [30]. Results were expressed as µg quercetin equivalents per mg dry extract.

### 2.4. Antioxidant Activity

Antioxidant activity was assessed using the 2,2-diphenyl-1-picrylhydrazyl (DPPH) radical scavenging assay [31]. A 0.1 mL volume extract (0.25, 0.50, 0.75, 1 mg/mL) was mixed with 2.9 mL of 3.9 mg/100 mL DPPH in 90% methanol, incubated in the dark for 30 min at 4 °C, and absorbance was measured at 517 nm. Scavenging capacity was calculated as:

$$DPPH\cdot inhibition\left(\%\right)=\frac{Absorbance\;blank-Absorbance\;sample}{Absorbance\;blank}\times 100$$

All colorimetric assays (TPC, TFC, protein content, and DPPH) were performed using a Thermo Spectronic HELIOS ε spectrophotometer (Thermo Fisher Scientific, MA, USA). All experiments were performed in technical triplicate. Ascorbic acid (250–1000 µg/mL) was used as a positive control.

### 2.5. Antifungal Activity Assay

Agar diffusion assays were used to evaluate antifungal activity against *Fusarium oxysporum* f. sp. *cubense* TR4 (Foc TR4). PDA plates (1% agar) were prepared with six wells containing algal extracts, DMSO controls, nystatin (17.5 µg; positive control), and an empty well (negative control). Extracts were prepared in DMSO (100 mg/mL), and 100 or 200 µL were added to each well, corresponding to doses of 10 and 20 mg per well, respectively. An 8 mm mycelial plug of Foc TR4 was placed at the center of each plate, and radial growth was recorded daily for 10 days.

### 2.6. HPLC-MS/MS Analysis

The compounds present in the methanol and ethyl acetate extracts were analyzed by high-performance liquid chromatography coupled to tandem mass spectrometry (HPLC–MS/MS) using an Agilent 1290 Infinity system coupled to an Agilent 6550 iFunnel Q-TOF mass spectrometer. Mass axis calibration was performed by continuous infusion of a reference solution consisting of 95% acetonitrile and 5% water. Chromatographic separation was carried out using a Zorbax Eclipse Plus C18 column (2.1 × 100 mm, 1.8 μm particle size). The injection volume was 1 μL, and the analysis was conducted at 4 °C over a 20 min run.

Data processing was performed using the Agilent MassHunter software suite. MassHunter Data Acquisition 10.1 was used to operate the LC/QTOF 6550 system, while MassHunter Qualitative Analysis B.10.0, together with MassHunter PCDL Manager B.08, were used for qualitative analysis of the data. Compound identification was considered tentative, based on database matching (METLIN Agilent compound database) and MS/MS spectra, and no confirmation with authentic standards was performed.

### 2.7. Statistical Analysis

Extraction yield was assessed with two-way ANOVA (species × solvent), while antioxidant capacity was evaluated by three-way ANOVA (species × solvent × extract concentration). Phenolic and flavonoid contents were analyzed by two-way ANOVA (species × solvent).

The antifungal activity was analyzed with generalized linear models (GLM, Gaussian family), including day and treatment as predictors and repeated measures as a random effect. Growth data at day 7 were further analyzed by one-way ANOVA (treatment). Post hoc tests included Tukey HSD for ANOVA and pairwise *t*-tests with Sidak correction (emmeans; [32]). Model assumptions were tested with Shapiro–Wilk and Levene’s tests; GLM validation was performed with DHARMa [33]. If parametric assumptions were not met, data were square-root transformed.

To integrate biological and chemical performance and prioritize the most promising algal extracts, a multiobjective Pareto analysis was applied.

This approach identifies extracts that achieve the best overall balance between antifungal activity, antioxidant potential, and extraction yield, allowing for a multidimensional comparison without imposing arbitrary weights.

Unlike PCA, which focuses on variance structure and dimensionality reduction, Pareto analysis directly identifies optimal candidates based on multiple criteria, making it more suitable for extract selection.

The analysis considered six maximization criteria: antifungal inhibition at 10 and 20 mg/well, DPPH scavenging activity at 1 mg/mL, total phenolic and flavonoid contents (µg GAE/mg and µg QE mg, respectively), and extraction yield (%).

Each extract (species × solvent) was represented as a point in this multidimensional space and classified as Pareto-optimal when no other extract outperformed it simultaneously in all variables. Pareto-optimal extracts thus define the non-dominated frontier, representing the most balanced and efficient candidates for downstream fractionation and valorization. All analyses and graphics were performed in R 4.3.3 [34].

## 3. Results

### 3.1. Identification of Algal Species

*Rugulopteryx okamurae* (E.Y. Dawson) I.K. Hwang, W.J. Lee and H.S. Kim, 2009, shows a yellow–brown thallus with dichotomous branching (isotomous and anisotomous), reaching up to seven branching orders. In the apical and medial zones, the medulla is observed to be single-layered medially and multistratified at the margins, with two to three marginal layers (Figure S1).

*Dictyota fasciola* (Roth) J.V. Lamouroux, 1809, exhibits a yellow–brown thallus, with lighter yellow coloration in apical regions. Branching occurs, forming dichotomies with two to four branching orders. Axes show a spiral arrangement and acute apical terminations. Both the medulla and marginal regions are unstratified in apical and medial zones (Figure S2).

*Codium fragile* (Suringar) Hariot, 1889, develops a dark green siphonous plectenchymatous thallus with dichotomous branching. Utricles are green, globose, and mucronate, lacking apical teeth (Figure S3).

*Batophora* sp. J. Agardh, 1854, displays a yellow–green thallus with darker green branches. The central cylindrical axis bears verticils of 7–11 branches, showing dichotomous and trichotomous branching up to seven orders (Figure S4).

*Palisada tenerrima* (Cremades) D. Serio, M. Cormaci, G. Furnari and F. Boisset, 2010, presents a green–brown thallus that turns violet in older basal branches. Attachment occurs through a basal disk (≈2 cm diameter). Branching follows three to four orders. Cells are irregularly polyhedral and slightly parallel in arrangement, with no *corps en cerise* or secondary pit connections. Transverse sections reveal elongated palisade-like cortical cells (Figure S5).

Molecular analysis confirmed the morphological identification for all species except for *C. fragile*, whose DNA was not successfully amplified (Table S1).

### 3.2. Chemical Characterization and Extraction Yield of the Studied Algae

All species showed a water content above 70%, with significant differences among them (ANOVA, F_4,28_ = 12.97, *p* < 0.001) (Figure S6A, Table S2). *C. fragile* showed the lowest value (79.7 ± 4.98%), whereas *D. fasciola* reached the highest (94.5 ± 2.49%).

Extraction yield depended on both solvent and species (two-way ANOVA, F_4,20_ = 26.97, *p* < 0.001) (Figure S6B, Table S3). Methanolic extracts generally produced higher yields than ethyl acetate extracts. Exceptions included *Batophora* sp., where the solvent had no significant effect. Among methanolic extracts, *D. fasciola* and *R. okamurae* achieved the highest yields (12.5 ± 0.41 and 12.4 ± 0.34), while ethyl acetate extraction favored *R. okamurae* (10.2 ± 0.52%). *Batophora* sp. and *P. tenerrima* produced the lowest yields overall.

**Figure 1:**
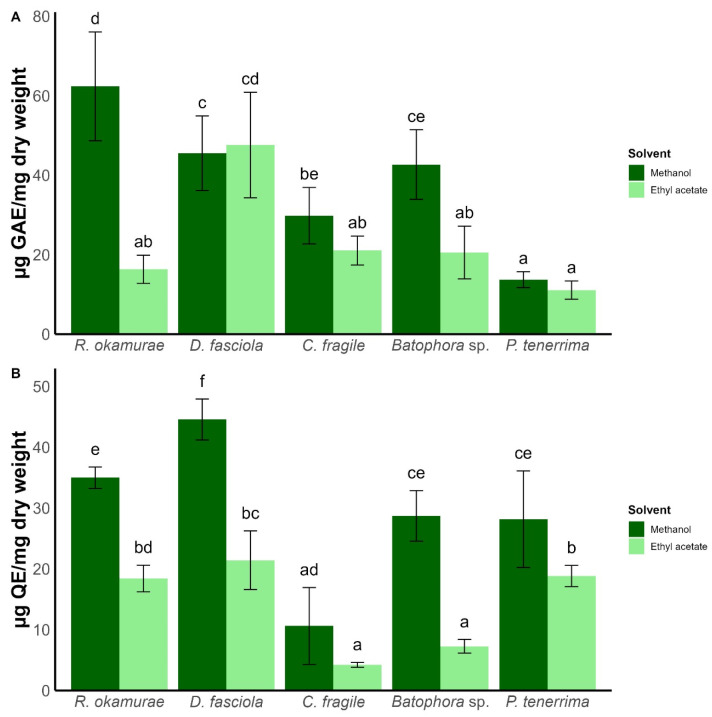
Phenolic (**A**) and flavonoid (**B**) content according to algae species and solvent used. Bars represent mean ± SD (n = 3). Different letters indicate statistically significant differences (two-way ANOVA followed by Tukey’s test, *p* < 0.05).

Total phenolic content varied significantly across species and solvents (two-way ANOVA, F_4,50_ = 17.04, *p* < 0.001) (Figure 1A, Table S4). Methanol consistently extracted higher levels of phenolics, particularly in *R. okamurae* (62.3 ± 13.7 µg GAE/mg). In contrast, *P. tenerrima* contained the lowest phenolic levels (13.7 ± 1.99 µg GAE/mg). Within ethyl acetate extracts, *D. fasciola* showed the highest phenolic concentration (47.6 ± 13.3 µg GAE/mg).

Flavonoid content also depended on solvent and species (two-way ANOVA, F_4,50_ = 9.68, *p* < 0.001) (Figure 1B, Table S5). Methanol extracts accumulated higher flavonoid concentrations than ethyl acetate, except in *C. fragile*, where flavonoid levels were similar in both solvents. The methanolic extract of *D. fasciola* reached the maximum (44.6 ± 3.37 µg QE/mg). Ethyl acetate extracts of *D. fasciola* and *R. okamurae* still surpassed 25 µg QE/mg, while *C. fragile* showed the lowest flavonoid content (4.24 ± 0.40 µg QE/mg).

### 3.3. Antioxidant Potential of Algal Extracts

The antioxidant activity of the extracts was influenced by species, solvent, and extract concentration (three-way ANOVA, F_15,96_ = 3.07, *p* < 0.001) (Table S6).

**Figure 2:**
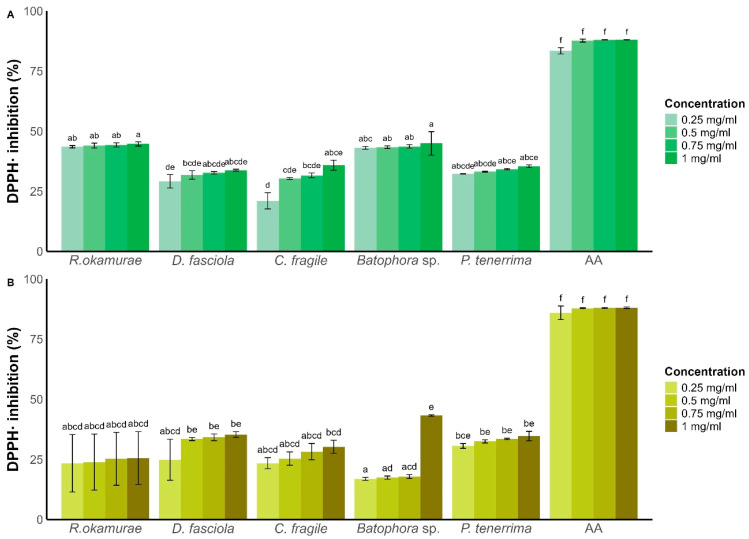
DPPH· radical scavenging activity (%) according to algae species and concentration for methanol (**A**,**B**) ethyl acetate extracts. AA: ascorbic acid. Bars represent mean ± SD (n = 3). Different letters indicate statistically significant differences (two-way ANOVA followed by Tukey’s test, *p* < 0.05).

For methanolic extracts (Figure 2A), antioxidant capacity increased with extract concentration, though this trend was only statistically significant for *C. fragile* (21 ± 3.38% DPPH inhibition at 0.25 mg/mL vs. 35.8 ± 2.08% at 1 mg/mL; Figure 2A). Among methanolic extracts, *Batophora* sp. exhibited the highest antioxidant activity (44.9 ± 4.89% at 1 mg/mL), closely followed by *R. okamurae* (44.68 ± 0.86% DPPH inhibition at 1 mg/mL). The remaining species showed DPPH inhibition below 40% at all tested concentrations.

For ethyl acetate extracts (Figure 2B), the highest antioxidant activity was observed for *Batophora* sp. extracts (43.19 ± 0.30% DPPH inhibition at 1 mg/mL). This extract showed a 25.32% increase in antioxidant capacity between 0.75 mg/mL and 1 mg/mL, without a clear linear dose–response pattern.

All other ethyl acetate extracts exhibited DPPH inhibition below 35%, with *R. okamurae* showing the lowest antioxidant activity (25.54 ± 10.94% at 1 mg/mL).

When comparing the antioxidant capacity at the highest concentration (1 mg/mL), methanolic extracts generally outperformed ethyl acetate extracts across all species—except for *Batophora* sp., where the ethyl acetate extract had superior antioxidant activity.

These results suggest that species with higher phenolic and flavonoid contents tend to exhibit stronger antioxidant activity, supporting the role of these compounds as major contributors to radical scavenging capacity.

### 3.4. Antifungal Activity

**Figure 3:**
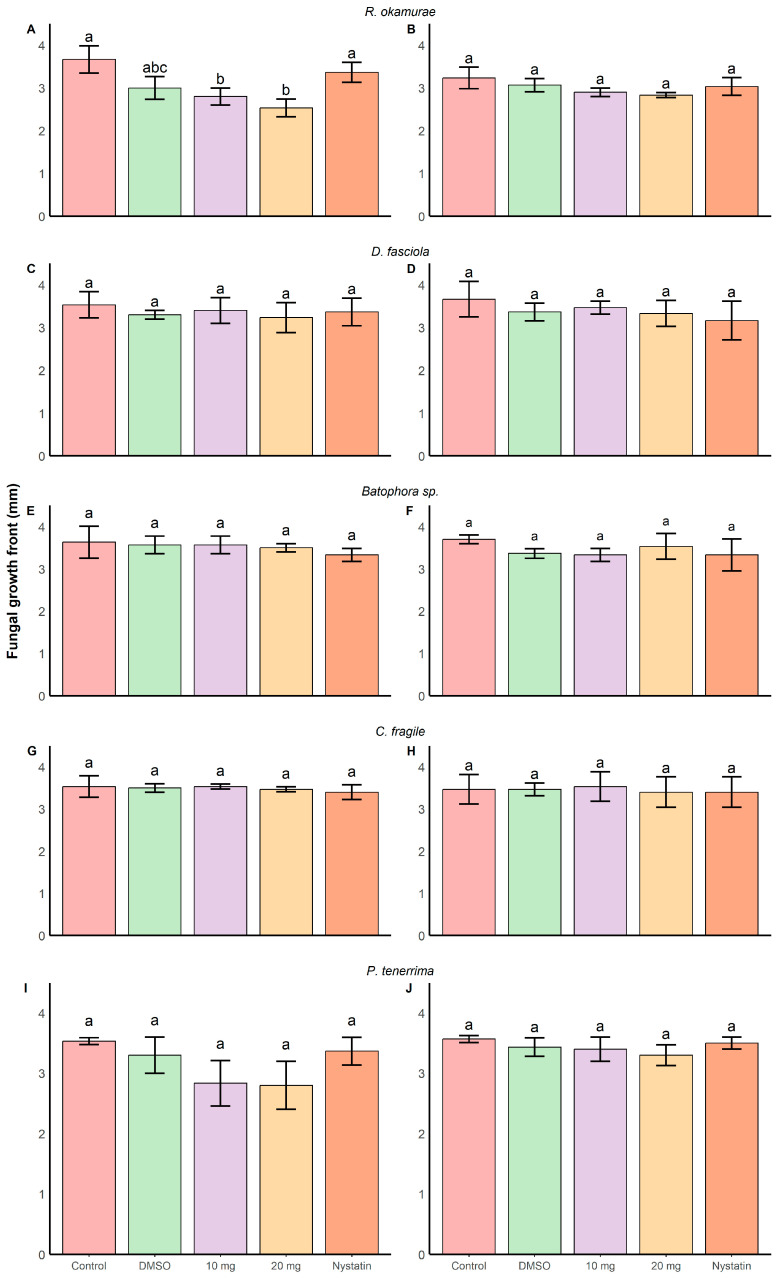
Effect of algal extracts obtained with methanol (**A**,**C**,**E**,**G**,**I**) and ethyl acetate (**B**,**D**,**F**,**H**,**J**) on the growth of *Foc* TR4. Panels correspond to extracts from *R. okamurae* (**A**,**B**), *D. fasciola* (**C**,**D**), *C. fragile* (**E**,**F**), *Batophora* sp. (**G**,**H**), and *P. tenerrima* (**I**,**J**). Bars represent mean ± SD (n = 3). Different letters indicate statistically significant differences between treatments (two-way ANOVA followed by Tukey’s test, *p* < 0.05).

Among all tested algal extracts, the methanolic extract of *R. okamurae* exhibited the strongest antifungal effect against *Fusarium oxysporum* f. sp. *cubense* TR4 (30.5 ± 9.13% inhibition relative to the control). A significant interaction was found between treatment and time (GLM, F_45,118_ = 1.58, *p* = 0.02) (Figure 3 and Figure S7, Table S7), indicating that fungal growth was reduced over time depending on the treatment applied. Biologically, this indicates that the antifungal effect is not only treatment-dependent but also time-dependent, suggesting a progressive inhibition of fungal growth rather than an immediate toxic effect. From day 6 onwards, 20 mg/well of extract significantly reduced colony expansion compared to the control and even to the commercial antifungal nystatin. This delayed response may indicate that the active compounds interfere with fungal growth processes over time, possibly affecting cell division or metabolic activity. At day 7 (D7) (Table S8), a significant treatment effect was also detected (ANOVA, F_5,12_ = 6.22, *p* < 0.001), with 20 mg of extract per well reducing growth to 2.53 ± 0.20 mm, compared to 3.66 ± 0.23 mm in the control.

Similarly, the methanolic extract of *P. tenerrima* significantly inhibited fungal growth (GLM, F_5,118_ = 24.68, *p* < 0.001), with both 10 and 20 mg/well doses showing inhibitory effects compared to the control (19.8 ± 10.4% and 20.7 ± 11.5% inhibition relative to the untreated control). However, post hoc comparisons at day 7 did not detect significant differences among treatments (ANOVA, F_5,12_ = 3.48, *p* = 0.03), although a reduction was observed (control: 3.53 ± 0.05 mm; 20 mg/well: 2.80 ± 0.40 mm; nystatin: 3.37 ± 0.23 mm). Although statistical significance was not reached in post hoc tests, the consistent reduction trend suggests a potential biological effect that may require higher replication or different assay conditions to be fully detected.

Other extracts showed more limited or inconsistent effects. The ethyl acetate extract of *P. tenerrima* showed no significant inhibition despite a statistically significant GLM result (F_5_,_118_ = 3.74, *p* = 0.03). Similarly, *D. fasciola* and *Codium fragile* extracts had variable outcomes: the ethyl acetate extract of *D. fasciola* significantly reduced growth (GLM, F_5,117_ = 8.27, *p* < 0.0001), but this effect was not significant at D7. The methanolic extract of *C. fragile* showed a significant treatment effect (GLM, F_5,118_ = 5.32, *p* < 0.001), but no individual treatment reached significance in post hoc tests. In contrast, the ethyl acetate extract of *C. fragile* at 10 mg/well did reduce fungal growth significantly.

*Batophora* sp. extracts displayed a mild and inconsistent antifungal effect. The methanolic extract showed no significant inhibition at any concentration, whereas the ethyl acetate extract at 20 mg/well slightly reduced fungal growth (GLM, F_5,118_ = 16.86, *p* < 0.001), though the effect was less pronounced than that of nystatin.

The solvent control (DMSO) did not show any significant effect on fungal growth compared to the untreated control at the doses used.

### 3.5. Multi-Criteria Optimization of Algal Extracts Through Pareto Analysis

**Figure 4:**
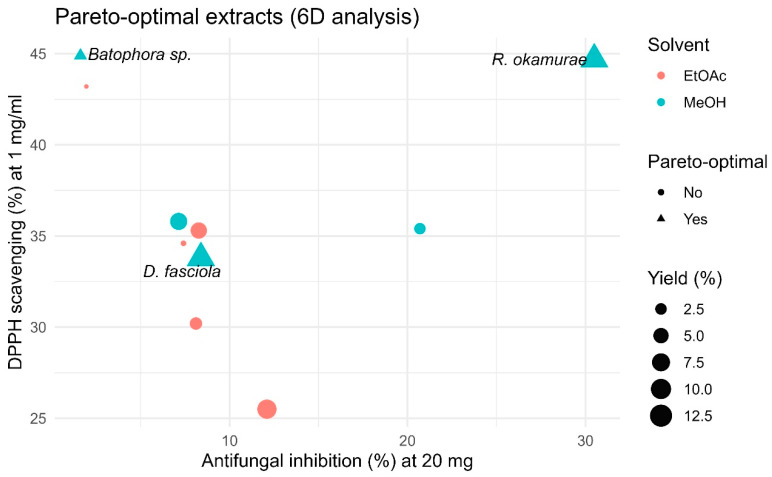
Pareto-optimal extracts (6D analysis). Scatter plot of antifungal inhibition at 20 mg/well (X) versus DPPH scavenging at 1 mg/mL (Y). Each point represents an extract (species × solvent combination). Point size denotes extraction yield (%); color indicates solvent (methanol, ethyl acetate). Triangles mark Pareto-optimal extracts computed over six maximization criteria (inhibition at 10 and 20 mg/well, DPPH, total phenolics, total flavonoids, and yield). Labels show *R. okamurae*, *D. fasciola*, and *Batophora* sp. as globally optimal candidates.

The five-dimensional Pareto analysis (criteria: antifungal inhibition at 10 and 20 mg/well, DPPH scavenging at 1 mg/mL, total phenolics, total flavonoids, and extraction yield) revealed a small subset of globally optimal extracts (Figure 4 and Table S9). In this approach, extracts are considered Pareto-optimal when no other extract performs better simultaneously across all evaluated criteria. Methanolic fractions of *R. okamurae*, *D. fasciola*, and *Batophora* sp. met this condition. This result highlights that the most promising extracts are those that combine multiple biological properties rather than excelling in a single parameter.

Among them, the methanolic extract of *R. okamurae* provided the best overall balance, combining the highest antifungal inhibition (~30% at 20 mg/well), strong antioxidant capacity (~45% DPPH), and the greatest extraction yield (~12%). *D. fasciola* (methanol extract) remained optimal through a balanced profile (moderate inhibition with comparatively high phenolic/flavonoid content and mid-range DPPH), whereas *Batophora* sp. (methanol extract) was mainly driven by exceptional antioxidant metrics (~45% DPPH) despite modest antifungal effects. All ethyl acetate extracts were dominated in the multidimensional space, underscoring the superior multifunctional performance of polar (methanolic) fractions.

### 3.6. HPLC-MS/MS Extract Analysis

Following the Pareto optimization, the methanolic extract of *R. okamurae* emerged as one of the most promising, combining high antioxidant activity, elevated phenolic content and strong antifungal effects against *Fusarium oxysporum* f. sp. *cubense* TR4. The ethyl acetate extract also showed relevant bioactivity in the Pareto analysis and was therefore included for comparative chemical profiling. To explore the chemical composition of both extracts, we used LC-MS.

Methanolic extracts showed by LC-MS a complex mixture of metabolites distributed across the retention time range (Tables S8 and S9). Accurate mass measurements and MS/MS fragmentation patterns indicated the presence of phenolic derivatives, oxylipin-type metabolites derived from C18 fatty acids, hydroxy fatty acids and terpenoid-like compounds. Several late-eluting signals matched sulfolipid derivatives commonly found in photosynthetic organisms. The analysis also revealed carotenoid pigments characteristic of brown macroalgae, including fucoxanthin and related xanthophyll derivatives.

We also found oxylipin-related compounds such as 12-oxophytodienoic acid (12-OPDA), likely from the oxidation of polyunsaturated fatty acids. The chromatographic profile also showed signals consistent with fucoxanthin, a carotenoid widely reported in brown algae. In addition, a peak detected at intermediate retention time corresponded to a compound with molecular formula C_15_H_22_O_2_, consistent with a bisabolene-type sesquiterpenoid related to curcumenone-like structures.

The LC–MS profile of the ethyl acetate extract showed a broadly similar but slightly less complex metabolite pattern. The signals mainly corresponded to terpenoid-like compounds, aromatic derivatives and lipid-related metabolites. Oxylipin-type compounds derived from polyunsaturated fatty acids also appeared in this extract, including signals compatible with 12-OPDA and related oxidation products.

Several peaks detected at intermediate retention times corresponded to compounds with molecular formulas compatible with sesquiterpenoid structures, including signals consistent with bisabolane-type compounds. Carotenoid pigments characteristic of brown macroalgae, including fucoxanthin, were also detected in this extract.

Overall, the ethyl acetate extract showed a lower diversity of polar metabolites compared with the methanolic extract, which may partly explain the lower antioxidant and antifungal activities observed in the bioassays.

## 4. Discussion

This study evaluated extracts from native and invasive macroalgae from the southeastern Spanish coast. These extracts displayed both antioxidant and antifungal activities.

Methanolic extractions consistently yielded higher recoveries than ethyl acetate, in line with previous reports showing that polar solvents, such as methanol, extract efficiently algal metabolites [35,36]. Our yields (≈2–12%) fit within the range reported for similar methods [37,38]. Differences with other studies may reflect solvent polarity, time of extraction, or diverse species composition [39,40]. The maceration method applied here also explains the lower yields compared to Soxhlet or other optimized techniques [41].

Phenolic and flavonoid contents varied among species. In *R. okamurae* and *D. fasciola*, values ranged from 17 to 60 µg GAE/mg, below the higher levels reported for other brown algae when using optimized methods [42]. However, they remain comparable to studies with similar maceration protocols [7,43]. Our *C. fragile* extracts showed lower phenolic and flavonoid levels than reported elsewhere [44], while *Batophora* sp. had fewer phenolics than related green algae [45]. For *P. tenerrima*, phenolic levels were in the same range as those of other *Laurencia complex* species [46].

Antioxidant activities from our extracts ranged between 20 and 50% DPPH inhibition, lower than in some *Dictyota* and *Codium* studies [47,48] but higher than reported for *Palisada* species [49]. The general trend (brown > green > red algae) agrees with previous observations [50,51] and reflects differences in algae metabolite composition. Brown algae are rich in terpenes and pigments such as fucoxanthin, both with strong antioxidant properties [52,53]. The relatively high antioxidant activity of *Batophora* sp. may relate to coumarins, previously described from Dasycladales algae [54].

Regarding antifungal capacity, brown algae again showed the highest inhibition of *Fusarium oxysporum* f. sp. *cubense* TR4, followed by moderate activity in the green alga *C. fragile* and the red alga *P. tenerrima*. Similar patterns have been attributed to phenolics and terpenoids in brown algae [55,56], which are known to exert antifungal effects through membrane disruption, increased permeability, and interference with fungal enzymatic systems in bacteria [57,58,59]. In *R. okamurae*, activity may also involve acidic vacuolar compounds, such as sulfuric acid [60], potentially contributing to local pH alterations that impair fungal growth. The Ceramiales, including *P. tenerrima*, produce halogenated sesquiterpenes and other terpenoids with reported antifungal properties, often associated with membrane destabilization and inhibition of key metabolic pathways [61,62,63]. In *C. fragile*, compounds such as diosgenin and saponins may contribute to antifungal activity by interacting with sterols in fungal membranes, leading to cell leakage and structural damage [64,65]. Altogether, these results suggest that the antifungal activity observed is moderate and likely mediated by multiple mechanisms acting in combination, although further studies will be required to confirm these effects experimentally. These results also highlight the predominance of polar bioactive molecules, since methanolic extracts consistently outperformed ethyl acetate.

Beyond direct effects on fungal cells, recent advances in plant–pathogen interaction studies have highlighted that plant defense responses are highly heterogeneous and cell-type-specific [66]. In this context, bioactive compounds such as those identified in macroalgae extracts may interact with these complex defense responses, opening new perspectives for their application in plant protection strategies.

In general, environmental and seasonal factors may further explain differences with previous studies, as metabolite profiles shift with location, season, and growth phase [67,68,69,70]. These variations are driven by changes in environmental conditions such as light intensity, temperature, salinity, and nutrient availability, which modulate algal metabolism. For instance, previous studies have reported strong seasonal variation in antimicrobial activity, with higher activity during colder or stress-associated periods and reduced activity during summer, when growth is prioritized. This pattern has been linked to changes in secondary metabolite production rather than general physiological status, reflecting a metabolic trade-off between primary growth and the synthesis of bioactive compounds.

These results indicate that several macroalgae with high representativity in the southeastern Spanish coast may be potential sources of antioxidant and antifungal compounds. In particular, the methanolic extract of *R. okamurae* showed the highest inhibition of *Fusarium oxysporum* f. sp. *cubense* TR4 among the tested extracts, together with relevant phenolic content and antioxidant capacity. LC–MS/MS analysis further revealed a chemically diverse metabolite profile, including phenolic derivatives, oxylipin-type compounds and terpenoid-related structures that may contribute to the found bioactivity. Antifungal results suggest potential for further exploration as candidates for managing Fusarium wilt in banana and other potential applications of algal extracts in plant health management. These findings support the potential of Mediterranean macroalgae as natural resources for antifungal and antioxidant applications, while also supporting the valorization of biomass from invaders such as *R. okamurae*.

## 5. Conclusions

Our algal extracts exhibited antioxidant and antifungal activity, with brown algae showing the strongest performance, likely due to their higher content of polar metabolites such as phenolics and flavonoids. Among the species tested, *R. okamurae* stood out, combining high extraction yield, phenolic content, antioxidant capacity, and antifungal activity against *F. oxysporum* TR4, positioning it as a promising candidate for agricultural and biomedical applications. Additionally, *Batophora* sp. showed notable antioxidant activity, supporting its potential as a source of bioactive compounds. These results support the valorization of invasive biomass—particularly *R. okamurae*—as a source of bioactive compounds with demonstrated antifungal and antioxidant activity, while contributing to the management of its ecological impact on Mediterranean coasts.

## Data Availability

The datasets generated and analyzed during the current study are available from the corresponding author upon reasonable request.

## Supplementary Materials

The following supporting information can be downloaded at: https://www.mdpi.com/article/10.3390/plants15101485/s1.
