# Supplementary material for: Screening Antifungal and Antioxidant Activity of Macroalgae from SE Spain Highlights the Invader Rugulopteryx okamurae
Source: Plants (Basel). 2026 May 13;15(10):1485. doi: 10.3390/plants15101485 (PMC13210778; doi:10.3390/plants15101485)
Supplement: Supplementary file 1 [file plants-15-01485-s001.zip › plants-4293281-supplementary.pdf]

Supplementary material

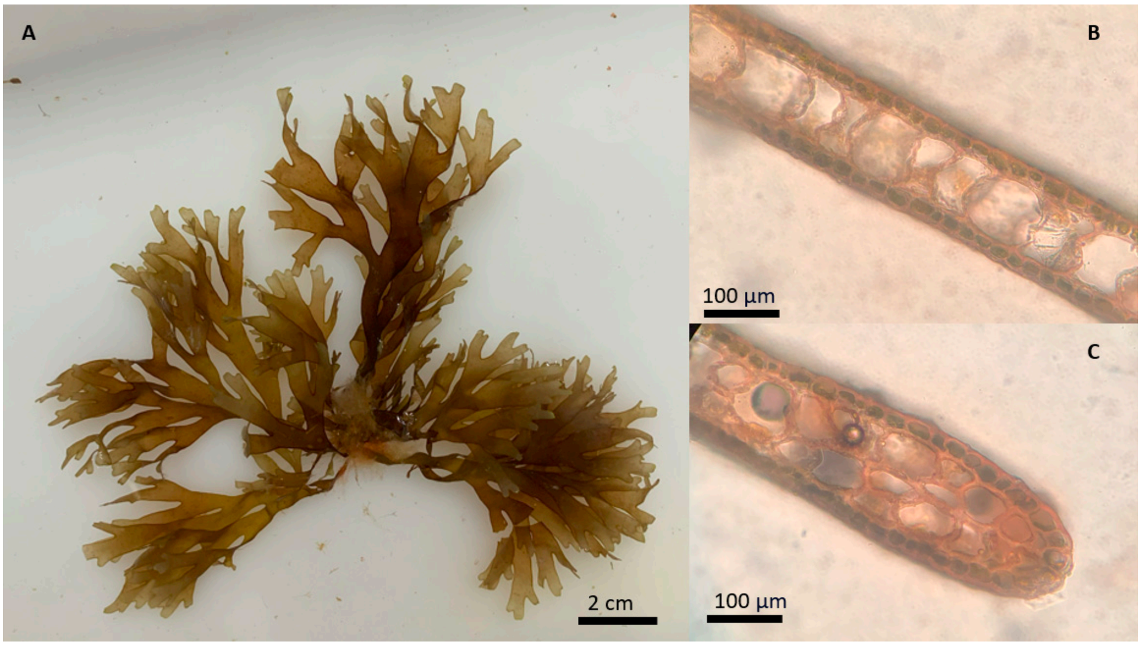

Figure S1. *Rugulopteryx okamurae*. General appearance of the alga (A); transverse section of the middle part showing the unistratified layer in the medial zone of the thallus (B); transverse section of the middle part showing the multistratified outer margin, with up to three cell layers (C).

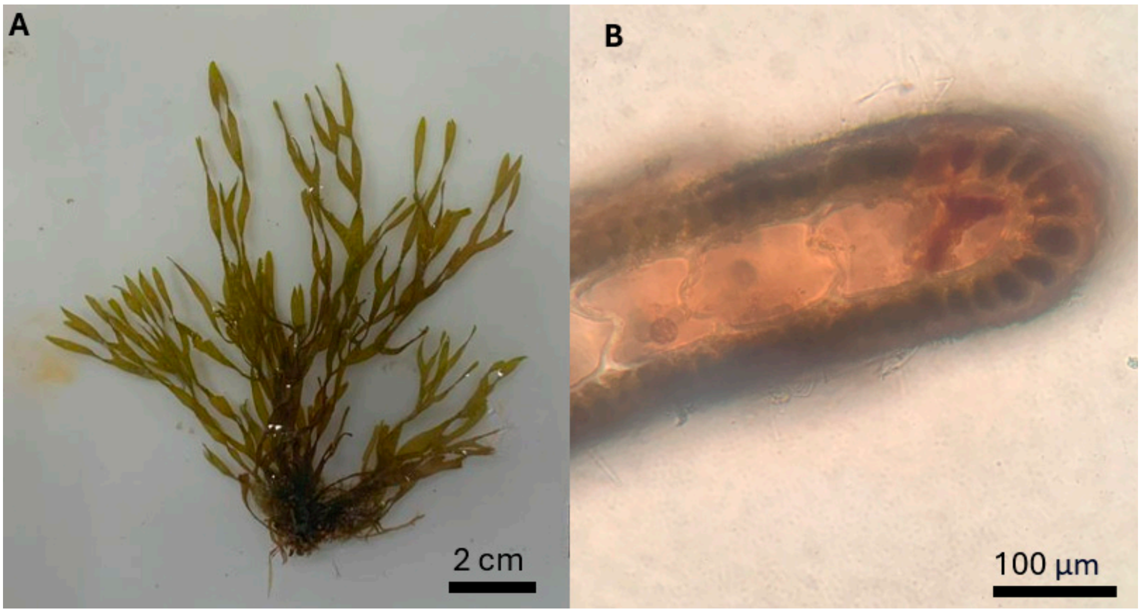

Figure S2. *Dictyota fasciola*. General appearance of the alga (A); transverse section of the middle part showing a unistratified medulla (B).

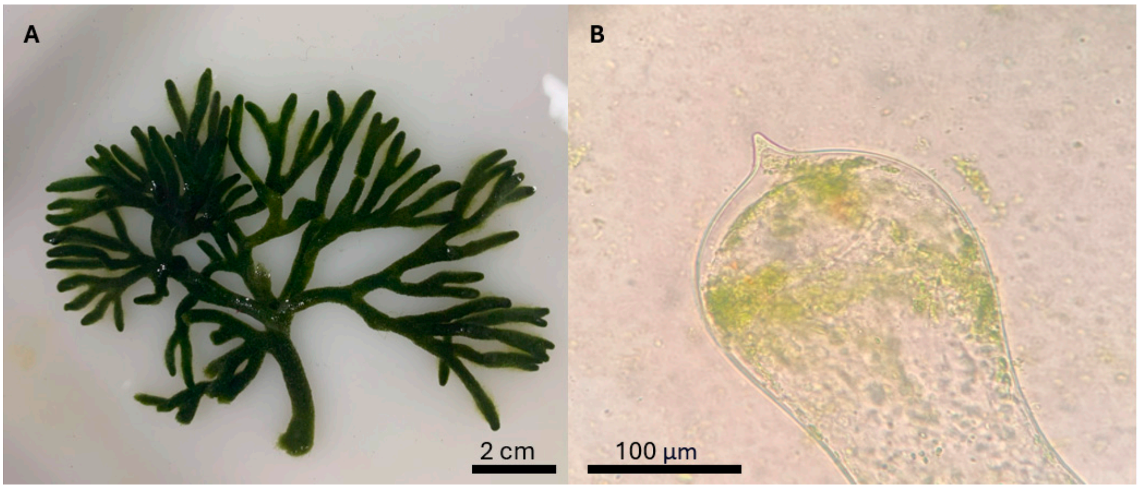

Figure S3. *Codium fragile*. General appearance of the alga (A); globose, mucronate utricles without corona (B).

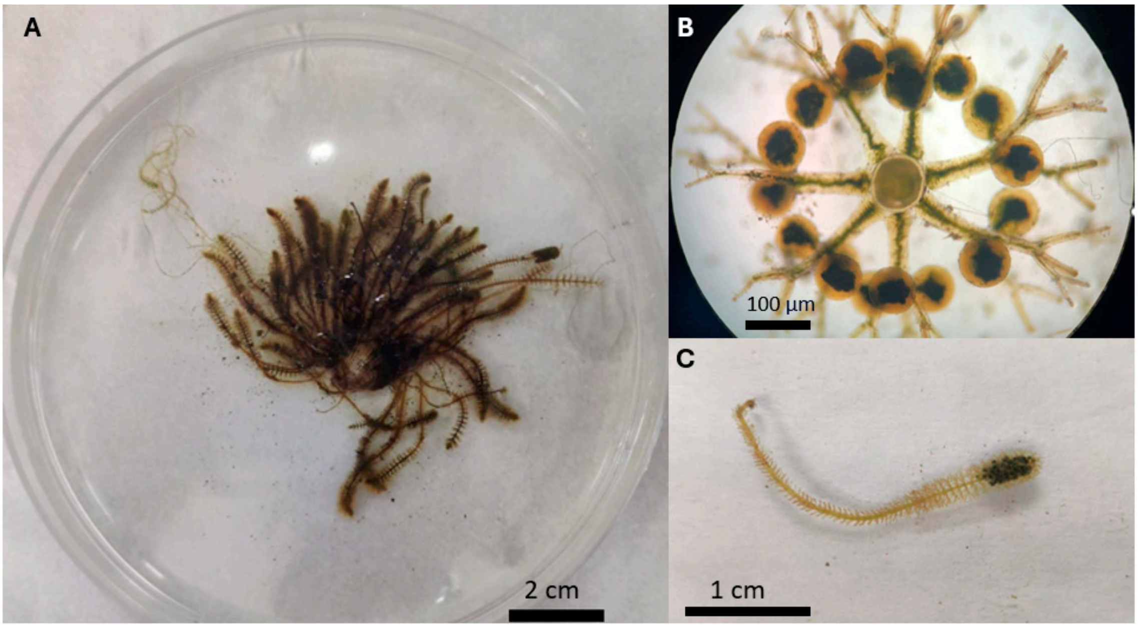

Figure S4. *Batophora* sp. General appearance of the alga (A); transverse section (B); detail of the branching pattern (C).

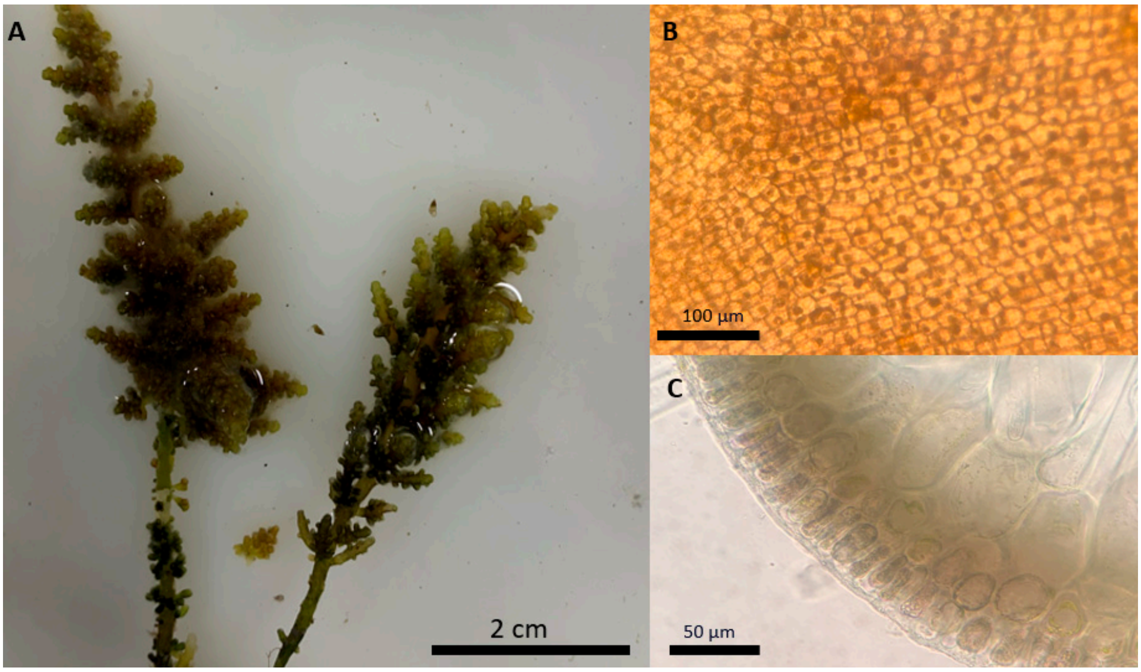

Figure S5. *Palisada tenerrima*. General appearance of the alga (A); surface view of the cells (B); transverse section of the middle part (C).

Table S1. Molecular identification of algal species included in this work. The top BLAST match is shown.

Algae	Marker	Identity	*Accesion number*
R. okamurae	psbA	98,73 %	MZ393490.1
	COI	100 %	GQ425120.1
D. fasciola	psbA	97 %	MW225012.1
Batophora sp.	rbcL	99,22 %	MH54529.1
P. tenerrima	COI	90,57 %	MG030786.1

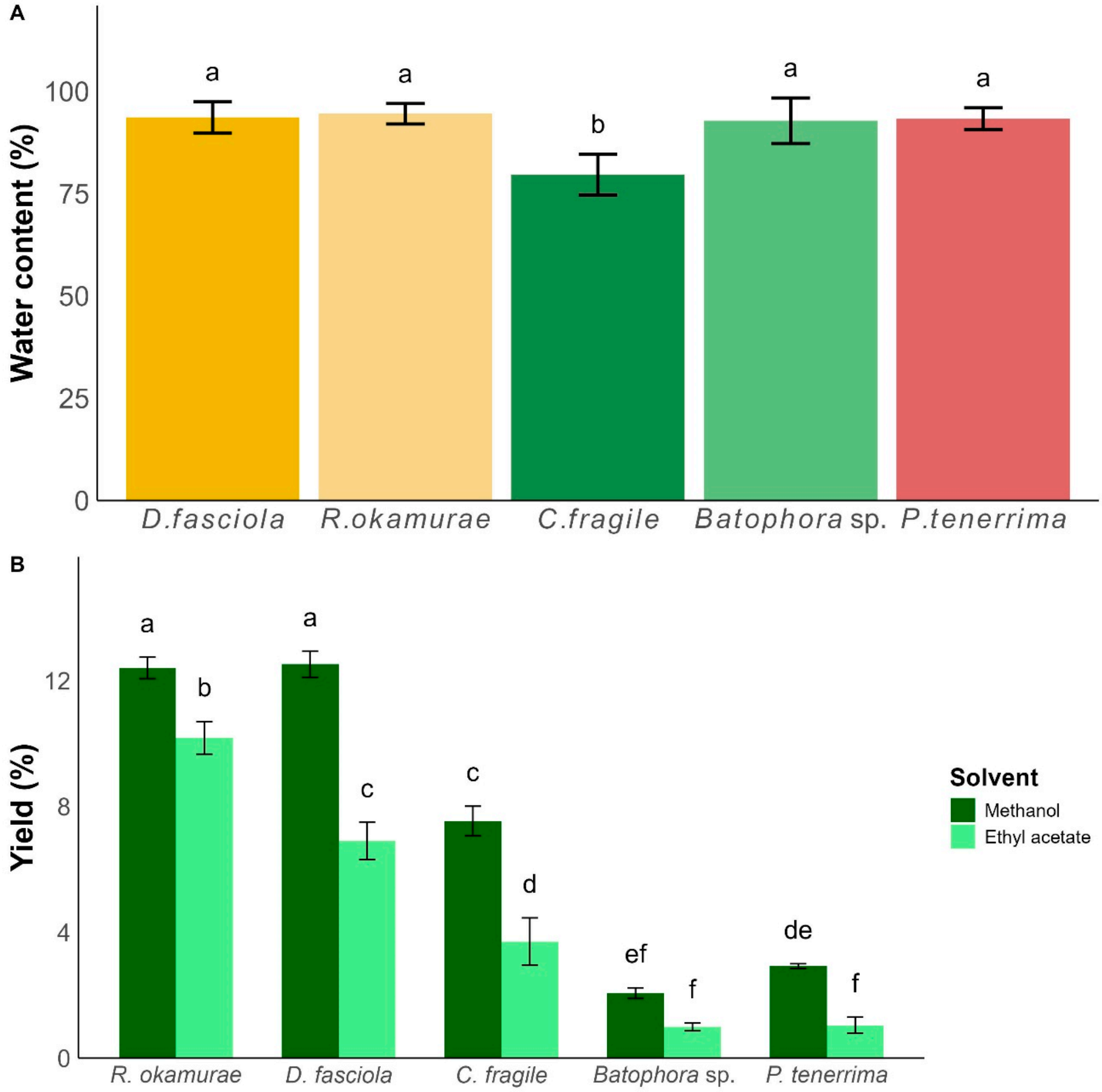

Figure S6. Characteristics of algal extracts used in this study. (A) Water content (%) of the different algae studied and (B) yield (%). Bars labeled with different letters indicate statistically significant differences ($p < 0.05$) according to the post hoc multiple comparison test. Bars sharing the same letter do not differ significantly from each other.

Table S2. ANOVA to assess water content differences among the studied algae. Df: degrees of freedom. Sum sq: sum of squares. Mean sq: mean squares.

	Df	Sum sq	Mean sq	F value	p.value
Algae	4	559.0	139.75	12.97	**<0.0001**
Residual	28	301.7	10.78		

Table S3. ANOVA to assess yield variation according to algae species and solvent used. Df: degrees of freedom. Sum sq: sum of squares. Mean sq: mean squares.

	Df	Sum sq	Mean sq	F value	p.value
Algae (A)	4	468.10	117.03	644.27	**<0.0001**
Solvent (S)	1	64.20	64.17	353.28	**<0.0001**
A x S	4	19.60	4.90	29.97	**<0.0001**
Residual	20	3.60	0.18		

Table S4. ANOVA to assess phenolic content variation according to algae species and solvent used. Df: degrees of freedom. Sum sq: sum of squares. Mean sq: mean squares.

	Df	Sum sq	Mean sq	F value	p.valor
Algae (A)	4	8262	2066	31.39	**<0.0001**
Solvent (S)	1	3605	3605	54.78	**<0.0001**
A x S	4	4484	1121	17.04	**<0.0001**
Residual	50	3290	66		

Table S5. ANOVA to assess flavonoid content variation according to algae species and solvent used. Df: degrees of freedom. Sum sq: sum of squares. Mean sq: mean squares.

Término	Df	Sum sq	Mean sq	F value	p.valor
Algae (A)	4	4482	1121	66.99	**<0.0001**
Solvent (S)	1	3548	3548	212.12	**<0.0001**
A x S	4	648	162	9.68	**<0.0001**
Residual	50	836	17		

Table S6. ANOVA to evaluate antioxidant activity (% DPPH· radical inhibition) as a function of algal species, solvent used, and concentration applied. Df: degrees of freedom. Sum sq: sum of squares. Mean sq: mean square.

Término	Df	Sum sq	Mean sq	F value	p.valor
Algae (A)	5	60648	12130	834.01	**<0.001**
Solvent (S)	1	1824	1824	125.42	**<0.001**
Concentration (C)	3	858	286	19.67	**<0.001**
A x S	5	2919	584	40.14	**<0.001**
A x C	15	660	44	3.03	**<0.001**
S x C	3	97	32	2.23	0,08
A x S x C	15	671	45	3.07	**<0.001**
Residual	96	1396	15		

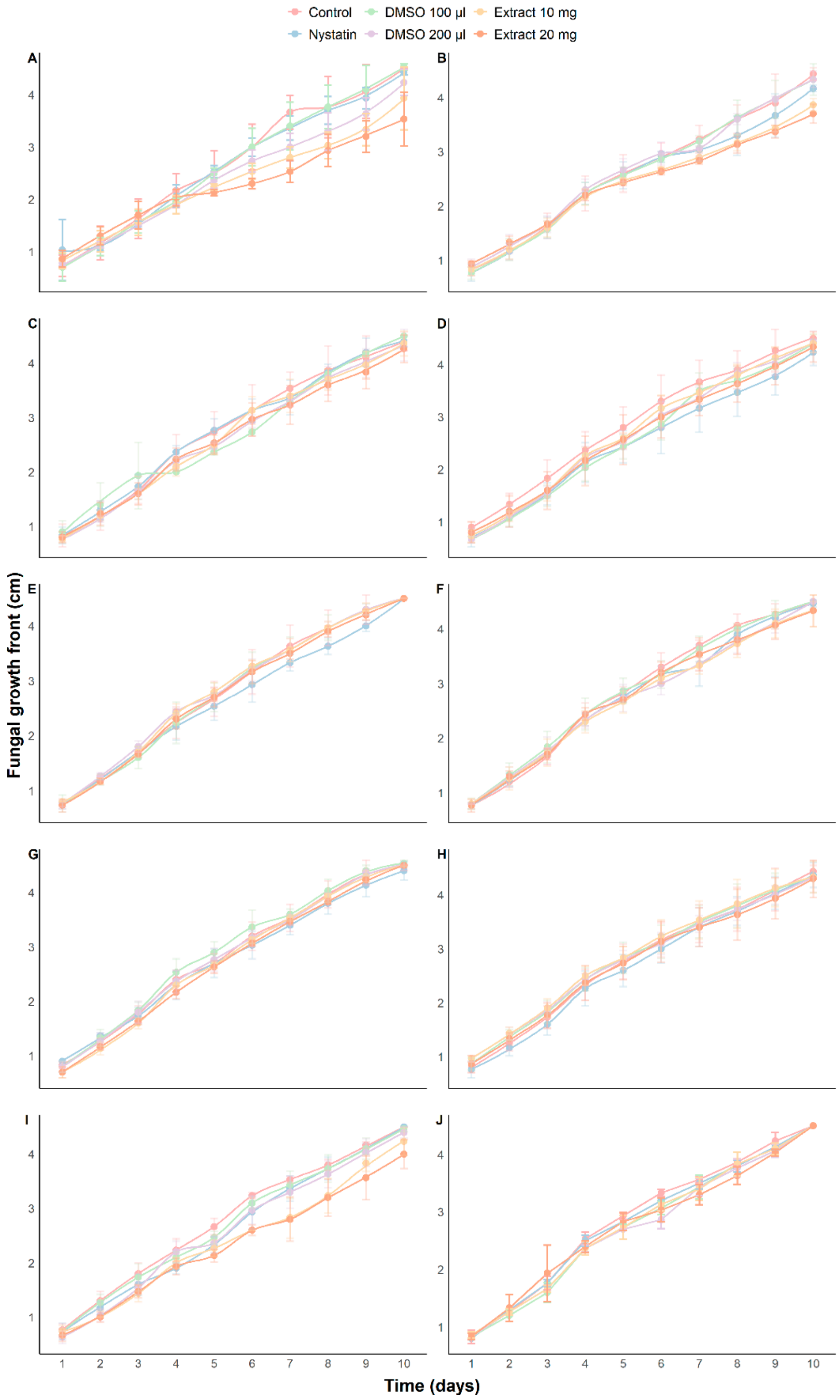

Figure S7. Effect of algal extracts obtained with methanol (left column) and ethyl acetate (right column) on the growth of banana wilt fungal pathogen *Fusarium oxysporum* f. sp. *cubense* Tropical Race 4 (FOC TR4). Panels correspond to extracts from *R. okamurae* (A–B), *D. spiralis* (C–D), *C. fragile.* (E–F), *Batophora sp.* (G–H), and *P. tenerrima* (I–J). Statistically significant differences (p < 0.05) are indicated by different letters.

Table S7. Type III analysis of variance using the Satterthwaite method for the linear mixed-effects model. The model evaluates the effect of treatment, day, and their interaction on fungal colony growth, with replication included as a random effect. **Sum sq**: sum of squares. **Mean sq**: mean squares. **NumDf**: numerator degrees of freedom. **DenDf**: denominator degrees of freedom.

	Sum sq	Mean Sq	NumDf	DenDf	F.value	p.value
R. okamurae methanol extract						
Treatment(T)	0.40	0.08	5	118	10.68	**<0.001**
Day (D)	23.18	2.57	9	118	338.98	**<0.001**
T x D	0.54	0.01	45	118	1.58	**0.02**
R. okamurae ethyl acetate extract						
T	0.13	0.02	5	118	8.14	**<0.001**
D	21.64	2.40	9	118	744.76	**<0.001**
T x D	0.18	0.004	45	118	1.29	0.13
D. fasciola methanol extract						
T	0.06	0.01	5	118	2.80	**0.01**
D	25.65	2.85	9	118	627.08	**<0.001**
T x D	0.14	0.003	45	118	0.71	0.90
D. fasciola ethyl acetate extract						
T	0.05	0.01	5	118	5.32	**<0.001**
D	28.96	3.21	9	118	1569.49	**<0.001**
T x D	0.05	0.001	45	118	0.56	0.98
C. fragile methanol extract						
T	0.05	0.10	5	118	4.75	**<0.001**
D	252.00	28.00	9	118	1291.81	**<0.001**
T x D	0.74	0.01	45	118	0.76	0.85
C. fragile ethyl acetate extract						
T	0.05	0.10	5	118	4.75	**<0.001**
D	252.00	28.00	9	118	1291.81	**<0.001**
T x D	0.74	0.01	45	118	0.76	0.85
Batophora sp. methanol extract						
T	0.07	0.01	5	118	8.40	**<0.001**
D	28.08	3.12	9	118	1692.06	**<0.001**
T x D	0.07	0.001	45	118	0.88	0.66
Batophora sp. ethyl acetate extract						
T	0.07	0.01	5	118	16.86	**<0.001**

D	23.83	2.64	9	118	2887.88	**<0.001**
T x D	0.05	0.001	45	118	1.39	0.07
P. tenerrima methanol extract						
T	0.44	0.08	5	118	24.68	**<0.001**
D	27.21	3.02	9	118	836.82	**<0.001**
T x D	0.13	0.003	45	118	0.84	0.72
P. tenerrima ethyl acetate extract						
T	0.03	0.006	5	118	3.74	**<0.01**
D	25.94	2.88	9	118	1673.75	**<0.001**
T x D	0.07	0.001	45	118	0.92	0.60

Table S8. ANOVA assessing the effect of treatments of the different extracts on the growth front of FOC TR4. Df: degrees of freedom. Sum sq: sum of squares. Mean sq: mean squares.

	Df	Sum sq	Mean sq	F value	p.value
R. okamurae methanol extract					
Treatment (T)	5	2.69	0.53	6.22	**0.004**
Residual	12	1.04	0.08		
R. okamurae ethyl acetate extract					
T	5	0.37	0.07	2.95	0.057
Residual	12	0.30	0.02		
D. fasciola methanol extract					
T	5	0.16	0.03	0.47	0.79
Residual	12	0.84	0.07		
D. fasciola ethyl acetate extract					
T	5	0.43	0.13	0.78	0.57
Residual	12	1.31	0.10		
C. fragile methanol extract					
T	5	0.16	0.03	0.61	0.69
Residual	12	0.63	0.05		
C. fragile ethyl acetate extract					
T	5	0.39	0.07	1.39	0.29
Residual	12	0.67	0.05		
Batophora sp. methanol extract					
T	5	0.06	0.01	0.69	0.63
Residual	12	0.24	0.02		
Batophora sp. ethyl acetate extract					
T	5	0.43	0.08	0.78	0.57
Residual	12	1.31	0.10		
P. tenerrima methanol extract					
T	5	1.49	0.29	3.48	**0.03**
Residual	12	1.02	0.08		
P. tenerrima ethyl acetate extract					
T	5	0.12	0.02	0.98	0.46
Residual	12	0.30	0.02		

Table S9. Multiobjective input matrix and Pareto classification for algal extracts (species × solvent). Values used in the 6-dimensional Pareto analysis (maximized criteria: antifungal inhibition at 10 and 20 mg, DPPH at 1 mg/ml, phenolics [µg GAE /mg], flavonoids [µg QE/mg], and yield [%]). "Pareto" marks non-dominated extracts (globally optimal across all criteria). Negative antifungal values indicate growth above the plate control on day 7 (no inhibition or slight stimulation). Abbreviations: MeOH, methanol; EtOAc, ethyl acetate.

Specie	Solvent	Antifungal (10 mg) (%)	Antifungal (20 mg) (%)	DPPH (%)	Phenols µg GA/mg	Flavonoids µg QE/mg	Yield (%)	Pareto
R. okamurae	MeOH	23.30	30.50	44.7	62.3	35.0	12.4	T
D. fasciola	MeOH	3.62	8.39	33.8	45.5	44.6	12.5	T
Batophora sp.	MeOH	-0.39	1.62	44.9	42.7	28.7	2.1	T
R. okamurae	EtOAc	10.10	12.10	25.5	16.3	18.4	10.2	F
D. fasciola	EtOAc	4.59	8.27	35.3	47.6	21.4	6.9	F
Batophora sp.	MeOH	-1.95	1.95	43.2	20.5	7.29	1.0	F
C. fragile	EtOAc	1.40	7.14	35.8	29.8	10.6	7.54	F
C. fragile	EtOAc	3.11	8.11	30.2	21.0	4.24	3.71	F
P. tenerrima	MeOH	19.80	20.70	35.4	13.7	28.2	2.93	F
P. tenerrima	EtOAc	4.60	7.41	34.6	11.1	18.8	1.05	F

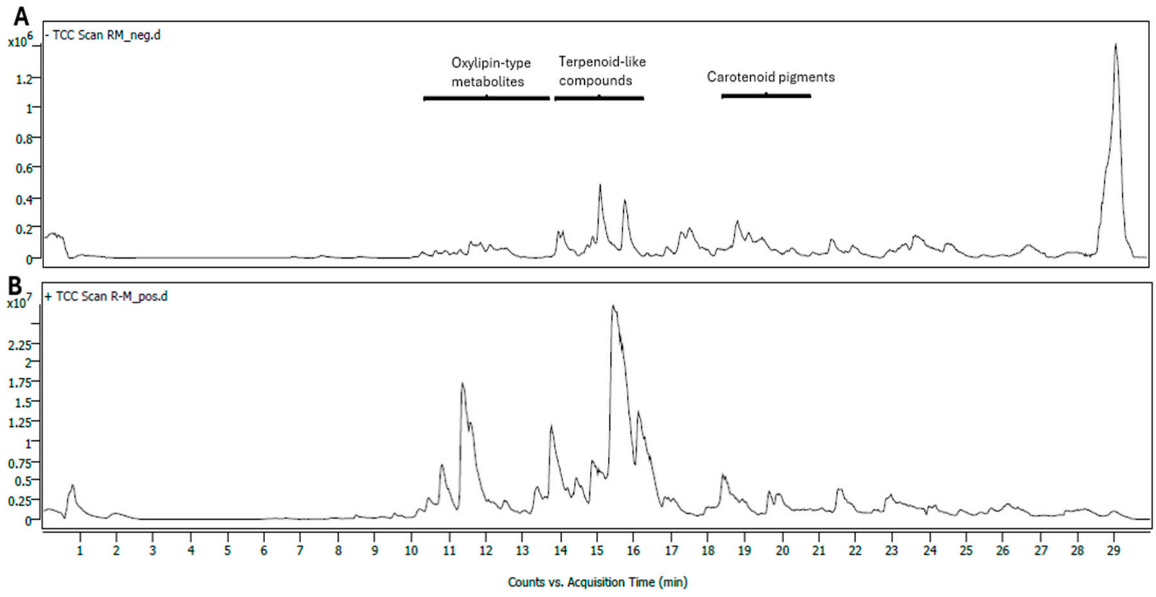

Figure S8. LC–MS chromatographic profiles of the methanolic extract of *R. okamurae* obtained in negative (A) and positive (B) electrospray ionisation modes. Annotated regions indicate approximate retention time ranges where oxylipin-type metabolites, terpenoid-like compounds and carotenoid pigments were detected based on accurate mass and MS/MS fragmentation data.

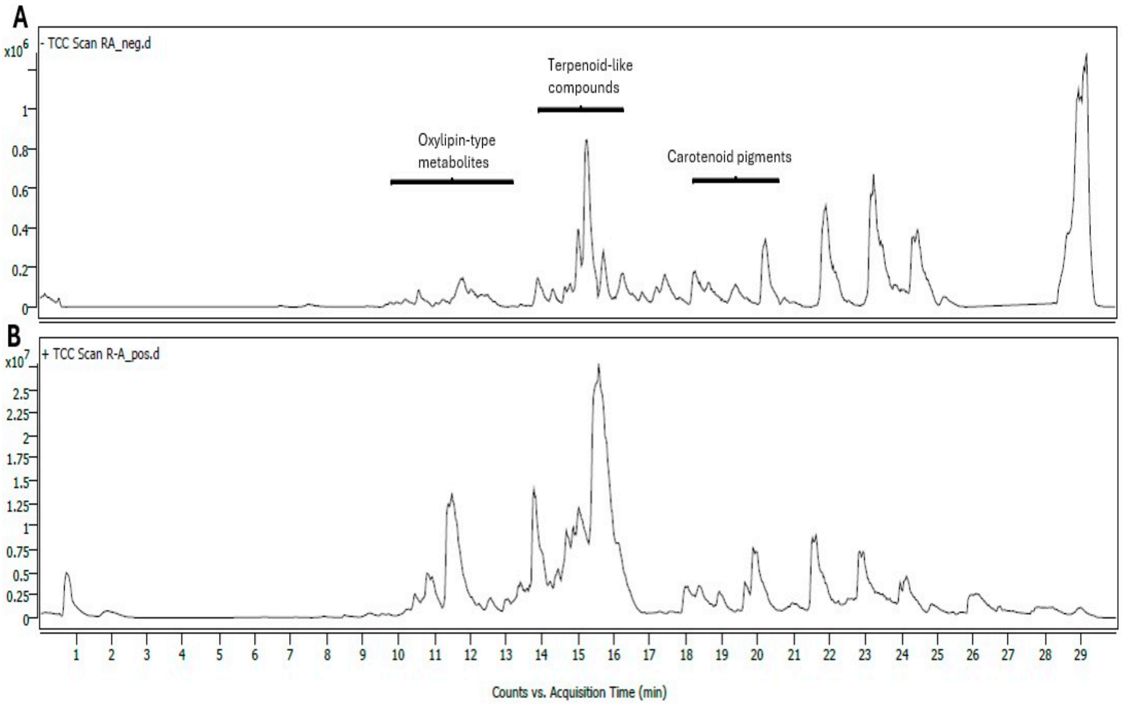

Figure S9. LC–MS chromatographic profiles of the ethyl acetate extract of *R. okamurae* obtained in negative (A) and positive (B) electrospray ionisation modes. Annotated regions indicate approximate retention time ranges where oxylipin-type metabolites, terpenoid-like compounds and carotenoid pigments were detected based on accurate mass and MS/MS fragmentation data.
